# Retraction notice to "Application of the unified method to solve the ion sound and Langmuir waves model" [Heliyon 8 (2022) e10924]

**DOI:** 10.1016/j.heliyon.2025.e43559

**Published:** 2025-06-28

**Authors:** Dulal Chandra Nandi, Mohammad Safi Ullah, Harun-Or Roshid, M. Zulfikar Ali

**Affiliations:** a Department of Statistics, Comilla University, Cumilla-3506, Bangladesh; b Department of Mathematics, Comilla University, Cumilla-3506, Bangladesh; c Department of Mathematics, Pabna University of Science and Technology, Pabna-6600, Bangladesh; d Department of Mathematics, University of Rajshahi, Rajshahi-6205, Bangladesh

This article has been retracted: please see Elsevier policy on article withdrawal (https://www.elsevier.com/about/policies-and-standards/article-withdrawal ).

This article has been retracted at the request of the Editor.

Post-publication, an investigation conducted by Elsevier's Research Integrity & Publishing Ethics team on behalf of the journal identified references that are irrelevant to the article. The authors were asked to comment upon the presence of these references in their work but were unable to satisfactorily address the reason for the references.

Additionally, the Elsevier's Research Integrity & Publishing Ethics team has determined that the authors were requested by two of the reviewers to insert redundant references to their paper during the peer-review process.

Consequently, the editor no longer has confidence in the integrity and the findings of the article and has decided to retract it. The scientific community takes a very strong view on this matter and apologies are offered to readers of the journal that this was not detected during the submission process.

The authors disagree with retraction and dispute the grounds for it.

